# Antihyaluronidase and Antioxidant Potential of *Atriplex sagittata* Borkh. in Relation to Phenolic Compounds and Triterpene Saponins

**DOI:** 10.3390/molecules28030982

**Published:** 2023-01-18

**Authors:** Karolina Grabowska, Wioleta Pietrzak, Paweł Paśko, Agnieszka Sołtys, Agnieszka Galanty, Paweł Żmudzki, Renata Nowak, Irma Podolak

**Affiliations:** 1 Department of Pharmacognosy, Medical College, Jagiellonian University, Medyczna 9, 30-688 Kraków, Poland; 2 Department of Pharmaceutical Botany, Medical University, 1 W. Chodźki, 20-093 Lublin, Poland; 3 Department of Food Chemistry and Nutrition, Medical College, Jagiellonian University, 9 Medyczna, 30-688 Kraków, Poland; 4 Department of Medicinal Chemistry, Medical College, Jagiellonian University, 9 Medyczna, 30-688 Kraków, Poland

**Keywords:** *Atriplex sagittata*, phenolic acids, flavonoids, LC-MS, saponins, antioxidant activity, hyaluronidase activity

## Abstract

The genus *Atriplex* provides species that are used as food and natural remedies. In this work, the levels of soluble phenolic acids (free and conjugated) and flavonoids in extracts from roots, stems, leaves and flowers of the unexplored *Atriplex sagittata* Borkh were investigated by LC-ESI-MS/MS, together with their antioxidant and antihyaluronidase activity. Phenolic acids were present in all parts of *A. sagittata*; and were most abundant in the leaves (225.24 μg/g dw.), whereas the highest content of flavonoids were found in the flowers (242.71 μg/g dw.). The most common phenolics were 4-hydroxybenzoic and salicylic acids, kaempferol-3-glucoside-7-rhamnoside, kaempferol-3-rutinoside and the rare narcissoside, which was present in almost all morphotic parts. The stem extract had the highest antioxidant activity and total phenolic content (611.86 mg/100 g dw.), whereas flower extract exerted the most potent antihyaluronidase effect (IC_50_ = 84.67 µg/mL; control—quercetin: IC_50_ = 514.28 μg/mL). Phytochemical analysis of the flower extract led to the isolation of two triterpene saponins that were shown to be strong hyaluronidase inhibitors (IC_50_ = 33.77 and 168.15 µg/mL; control—escin: IC_50_ = 307.38 µg/mL). This is the first report on the presence of phenolics and saponins in *A. sagittata.* The results suggest that both groups of metabolites may contribute to the overall activity of this plant species.

## 1. Introduction

The genus *Atriplex* (Amaranthaceae) comprises about 260 species of large herbaceous plants widespread throughout the world, especially in the arid and semi-arid regions of Europe, Asia, Africa, Australia, and North America [1]. The genus represents one of the oldest wild edible products; for example, species such as *A. hortensis*, *A. partulacoides*, and *A. sagittata* have been used for many centuries as a leafy vegetable [2,3,4,5]. Recent studies have shown that due to their high nutritional value and protein content, *A. hortensis* seeds can be a substitute for widely used quinoa seeds or cereal grains [4]. Some species, such as *A. portulacoides*, are suggested by some authors as functional food [5]. This pro-health approach is only partially a new concept considering that many species of the genus *Atriplex* have been valued in traditional and folk medicine. For example, *A. halimus* is listed among plant remedies for breast cancer [6], being also applied in diabetes [7], as a laxative and to relieve stomach pain [8]. *A. crossifolia* has been used to cure jaundice [9], while *A. hortensis* leaves are valued as a diuretic and purgative agent [3]. In turn, the entire *A. sagittata* plant has been recommended for diabetes [10].

Modern biological activity studies have shown that extracts from *Atriplex* species exert a wide range of activities, including antioxidant [11,12,13,14], anticholinesterase [13], antibacterial [15], antifungal [16] and antiparasitic activity [17]. Recent in vivo studies revealed an antidiabetic [18], a hepatoprotective [14] and a nephroprotective effect [19]. These activities are attributed to the presence of various bioactive compounds in *Atriplex* species such as triterpenes [20], sterols and phytoecdysteroids [21,22,23], but most importantly—triterpene saponins [24,25,26] and phenolic compounds [11,12,13,14,17,18,19,27,28]. Saponins represent an important group of plant metabolites that are characterized by a wide range of pharmacological activities and clinical applicability. Among the many uses of saponin-rich plants, their expectorant, anti-inflammatory, and antihyaluronidase effects are most valued, with glycyrrhizin, asiaticoside, or escin being some prominent examples [29,30,31]. Triterpene saponins have also been reported in some of the *Atriplex* species, such as *A. semibaccata* [26], *A.nummularia* [32], *A. glauca* [25], *A. lasiantha* [24], *A. tatarica* [28], *A. leucoclada* and *A. stilosa* [33,34].

In addition to saponins, phenolics are also important from a biological and nutritional point of view. Due to their antioxidant properties, they are used as natural preservatives of foods [35]. They also show a unique array of other biological activities: anti-inflammatory, antipyretic, antihyaluronidase, antirheumatic, antibacterial, antiviral, immunomodulatory, hepatoprotective, neuroprotective [36,37,38,39]. Among them, flavonoids are the most studied group of metabolites. Phytochemical studies revealed different types of flavonoids in species of the genus *Atriplex*, including common flavonols such as quercetin, kaempferol, and their glycosides [8,40], but also the more rarely found patuletin glycosides or sulphated flavonoids [41,42]. In turn, phenolic acids, which are otherwise not uncommon in other members of the Amaranthaceae family, have been reported so far in a limited number of species, for example, *A. lindley* [8], *A. semibacata* [43], *A. hortensis* [44], *A. halimus* [17,45,46], *A. lasiantha* [15], *A. mollis* [47], *A. portulcoides* [48]. However, the distribution of phenolic acids in individual parts of *Atriplex* plants has not been investigated so far.

Phenolic acids occur in plants in various forms, such as soluble free acids, soluble conjugated forms (glycosides, esters) and insoluble bound complexes. Plant extracts, prepared with an organic solvent, contain only extractable phenolic acids, thus soluble fractions (free and conjugated forms) [49,50,51].

Until now quantitative studies on phenolic acids have been performed on extracts from only a few species of the genus (*A. mollis*, *A. lindley*, *A. halimus*, *A. hortensis*, *A. portulcoides*, *A. lasiantha*) [8,15,44,46,47,48] and all published papers relate to the analysis of free phenolic acids in organic extracts. Some recent articles also report the presence of compounds from the conjugated phenolic acid group in *Atriplex* species (*A. halimus*, *A. gmelini*) [45,52,53].

Therefore, in view of the above data on the *Atriplex* genus, and the lack of phytochemical and biological studies on *Atriplex sagittata* Borkh. despite its use for both curative and food purposes [2,10], we decided to investigate this hitherto unexplored but widely distributed and easily available plant species.

The main objective of the current study was to identify and quantify soluble phenolic acids (free and conjugated) as well as flavonoids, in methanolic extracts from different morphotic parts of *A. sagittata* plant (roots, stems, leaves, flowers) together with the estimation of the total phenolic content (TPC) and the antioxidant activity. In addition, the antihyaluronidase activity of the extracts was investigated in relation to the phenolic compounds that occur in them. As the inhibitory activity against the enzyme that degrades hyaluronic acid is well documented not only for phenolics but also for saponins [54], the second objective of the present study was to investigate the presence of saponins in *A. sagittata* and to evaluate whether isolated saponins themselves will exhibit an antihyaluronidase effect and thus contribute to the activity of extracts.

## 2. Results and Discussion

### 2.1. LC-ESI-MS/MS Profile of Phenolic Acids

In the first phase of the study, a qualitative and quantitative analysis of the individual phenolic compounds was performed using LC-ESI-MS/MS in the extracts obtained from various morphotic parts of *A. sagittata*. The results are shown in Table 1.

Quantitative analysis showed that the highest level of free phenolic acids was found in *A. sagittata* flowers (45.20 μg/dw.) and leaves (43.84 μg/dw.). The most abundant phenolic acid in the soluble fraction of the investigated extracts was ferulic acid, followed by *p*-hydroxybenzoic and salicylic acid (Table 1). The concentrations of other free phenolic acids, such as protocatechuic, gentisic, 4-hydroxycynamic acid, syringic and 3-hydroxycynamic acids, which were detected in the samples, were lower than the limit of quantification (<LOQ). The phenolic profile differed in the analyzed plant parts. Ferulic acid was found exclusively in extracts of leaves (22.59 μg/g dw.) and flowers (18.57 μg/g dw.), whereas *p*-hydroxybenzoic and salicylic acids were present in all parts of the plant. Overall, the qualitative profile of phenolic acids in *A. sagittata* was consistent with the results obtained for other *Atriplex* species [8,17,44,45,46,47,48].

It is noteworthy that the presence of free gentisic acid in the flower and stem of *A. sagittata* reported in the current study, is the first information on this phenolic compound in the genus *Atriplex*. However, its content was too low to be quantified in the extracts. Furthermore, none of the samples of *A. sagittata* contained veratric, rosmarinic, or gallic acids. Although free gallic acid has been reported in some species of *Atriplex* (*A. halimus*, *A. portulacoides*, *A. mollis, A. lindleyi*) [8,17,46,47,48], studies published so far indicate that the presence of this phenolic acid depends greatly on the plant habitat [46] and the extraction method [47].

In addition to free phenolic acids, soluble conjugated phenolic acids were also quantified in the current investigation. To our knowledge, this study is the first report on the quantitative determination of conjugated compounds, not only in *A. sagittata* but also in any of the *Atriplex* species.

The results of the current study showed that conjugated soluble phenolic acids were present in much higher amounts than free compounds in each of the analyzed extracts from different plant parts of *A. sagittata* (Table 1), however, there were significant qualitative and quantitative differences. The highest content was found in the extract of leaves (225.24 μg/g dw.), followed by flowers (115.53 μg/g dw.). Lower levels were recorded in the roots and stems (Table 1). Generally, ten conjugated phenolic acids were present in the leaves, six in the flowers, five in the roots, and three in the stems were quantified. For conjugated forms in leaves and flower extracts, the predominant phenolic acid was 4-hydroxybenzoic acid (84.29 μg/g dw. and 52.12 μg/g dw., respectively). In leaf extract, gentisic and syringic acids were also present in significant amounts (Table 1). The latter was also the dominant conjugated acid in stems and roots. It is worth mentioning that these acids, although dominant in the conjugated form, were also present as free phenolic acids.

Ferulic acid was present in *A. sagittata,* both in free and conjugated form, but the results of our research indicate that, contrary to the above-mentioned acids, its free form probably predominates, mainly in leaves and flowers. However, the low content of this acid in the flower hydrolyzate may indicate its degradation during acid hydrolysis. Such a partial loss of ferulic acid has already been described in the literature [55].

Salicylic acid was also present in *A. sagittata* in free and conjugated form (Table 1). In extracts of leaves, roots, and stems, it dominated in a conjugated form. In turn, the difference between the salicylic acid content in the flower extract and its hydrolyzate was statistically insignificant, indicating that this phenolic acid was present in the flowers of *A. sagittata* mainly in the free form.

Furthermore, vanillic and caffeic acids, which were not found in the free form (<LOD) in all plant parts of *A. sagittata*, were detected and quantified in the analyzed samples of hydrolyzed extracts. The results obtained indicate that caffeic acid was present in conjugated form in the leaves, whereas vanillic acid occurred in all morphotic parts except the stem. Similarly, protocatechuic acid, absent in the free form in leaves and present, but in negligible concentration, in flower extracts, occurs in a significant amount in conjugated form in these parts of *A. sagittata*. It is worth noting that conjugated derivatives of protocatechuic acid (glucose protocatechuic acid, xylose protocatechuic acid) and caffeic acid (3,5-dicaffeoyl-*epi*-quinic acid; caffeic acid sulfate ester) have previously been identified in aerial parts of species of *Atriplex* such as *A. halimus* [45] and *A. gmelini* [52,53].

### 2.2. LC-ESI-MS/MS Profile of Flavonoids

The results of the flavonoid analysis are presented in Table 2. In different morphological parts of *A. sagittata* nine flavonoid glycosides were found. All have been detected in flowers. Except for naringenin and isovitexin/vitexin other flavonoids were detected in all parts of the plant (narcisoside was absent only in roots). The most frequent were kaempferol-3-glucoside-7-rhamnoside, kaempferol-3-O-rutinoside, and narcissoside, which were present in quantifiable amounts in all above ground morphotic parts. In general, less flavonoids were found in the underground parts. Although six different flavonoids have been identified in roots, their concentrations were lower than the limit of quantification. The highest level of flavonoid glycosides was found in flowers (242.71 μg/g dw.) and leaves (202.86 μg/g dw.). The most abundant compounds were isoquercetin in flowers (100.84 μg/g dw.), astragalin in leaves (77.38 μg/g dw.), and kaempferol-3-glucoside-7-rhamnoside in flowers and leaves (73.76 μg/g dw. and 97.13 μg/g dw.).

This is the first report to investigate the presence and content of flavonoids in *A. sagittata*. The qualitative profile is consistent with the data obtained for other representatives of the *Atriplex* genus, although it should be noted that the flavonoid profiles for individual species of the genus differ greatly. Rutin and naringin were detected in aerial parts of *A. mollis* [47], *A. tatarica,* and *A. verrucifera* [56], while isoquercetin was found in *A. lindleyi* [57]. Isorhamnetin glycosides, such as narcissoside and isorhamnetin-3-*O*-glucoside, were identified in *A. halimus* [27,46] and *A. farinosa* [58]. Astragalin was isolated from aerial parts of *A. semibaccata* [43]. Although there are data on the occurrence of kaempferol derivatives in some *Artiplex* species [40,41,43], to our knowledge, glycosides detected in the current study in *A. sagittata*, such as kaempferol-3-rutinoside and kaempferol-3-glucoside-7-rhamnoside, have not been identified so far. Nevertheless, it should be noted that recently Tran et al. [59] tentatively detected by LC/MS /MS, the presence of kaempferol-rhamnosyl-glucoside in extracts of *A. hortensis*. Furthermore, there are also no reports indicating the presence of apigenin derivatives such as isovitexin or vitexin in *Atriplex* species, so the results of our study on *A. sagittata* provides novel information.

### 2.3. Antioxidant Activity of Extracts

Although there are many reports on the antioxidant potential of extracts from various species of the genus *Atriplex* [11,13,14,15,40,60,61], none of them concern *A. sagittata*. To evaluate the antioxidant activity of methanolic extracts from different morphological parts of *A. sagittata*, two methods were used: DPPH radical scavenging and FRAP activity.

The FRAP method is based on determination of the ferric-tripyridyltriazine complex reducing ability. Because of the involvement of metal ions in the analytical reaction, this method is fast, sensitive, and spans a relatively wide range of antioxidant substrates. However, the results obtained using the FRAP method express the corresponding concentrations of electron-donating antioxidants. Thiol antioxidants and carotenoids that act by quenching radicals cannot be determined by this assay. The DPPH method is based on the evaluation of the reducing capacity of antioxidants toward a stable nitrogen radical, possessing an odd electron. The color of its solution disappears rapidly when it encounters radical scavengers, and steric accessibility is a major determinant of this reaction. Therefore, this assay is adequate mainly for reactive small molecules that have good access to the radical site and is less sensitive for larger molecules. The DPPH assay is more appropriate for samples with lipophilic antioxidants or those with a high lipid content. It should be noted that the potential for interaction/polymerization of phenolic compounds can affect antioxidant ability which is often underestimated in natural product samples. Thus, no single antioxidant assay method can deliver a full antioxidant capacity of natural bioactive compounds that show complex kinetics [62,63,64].

The results of the antioxidant activity tests for *A. sagittata* are shown in Table 3. The stem extracts of *A. sagittata* showed significantly higher antioxidant activity in the FRAP and DPPH tests (FRAP 5.46 mmolFe^2+^/100 g dw.; DPPH 2.99 mmolTrolox/100 g dw.) compared to extracts prepared from other parts of the plant (Table 3). Generally, extracts can be ranked according to decreasing antioxidant activity as follows: stem extract > leaf extract > flower extract ≈ root extract. According to Kachout [65], antioxidant activity is very important for the adaptive ability of plants of the *Atriplex* genus, as it protects them against environmental stress.

Numerous studies show that both flavonoids and phenolic acids exhibit potent antioxidant activity [66,67,68,69,70]. Surprisingly, in the current study, no correlation was found between the content of the sum of flavonoids or phenolic acids and the antioxidant activity. However, a relationship was observed between antioxidant potential and total phenolic content (TPC). The highest total phenolic content (TPC) was observed for the stem, followed by leaves, flowers, and roots (Table 3). In no current study, a correlation was observed between the TPC level and the sum of quantified phenolic acids or flavonoids. Therefore, our research suggests that in addition to the identified and quantified phenolic acids and flavonoids, other unidentified phenolic components are present in *A. sagittata* extracts, which may affect the biological activity of plant extracts. This issue requires further in-depth research.

### 2.4. Antihyaluronidase Activity of Extracts

This is the first report on the antihyaluronidase activity of any *Atriplex* species. Several reports indicate that inhibition of hyaluronidase is associated with phytochemicals belonging to phenolic compounds [54,71,72]. The results of the hyaluronidase inhibitory assay are shown in Table 4.

The study revealed that all extracts tested from different morphotic parts of *A. sagittata* were potent inhibitors of hyaluronidase activity and affected the enzyme dose-dependently. It is worth noting that their activity was much higher compared to the positive control, quercetin (IC_50_= 514.28 µg/mL), which is a well-known hyaluronidase inhibitor [54].

Interestingly, the flower extract, which was characterized by the highest content of flavonoids and free phenolic acids (Table 1 and Table 2), demonstrated the highest antihyaluronidase activity (IC_50_ = 84.67 µg/mL). However, no correlation was observed between antihyaluronidase activity of any of the extracts tested and their TPC level, the sum of phenolic acids and flavonoids, or the contents of individual phenolic acids and flavonoids. This suggests that the observed activity may be related not only to phenolics and flavonoids but also to other compounds or to the synergism of action.

Published data indicate that not only phenolic compounds but also other plant metabolites, such as saponins, can inhibit the activity of hyaluronidase. Furthermore, some saponins, such as escin, are recommended as a drug with anti-inflammatory and antihyaluronidase potential [54]. Taking into account reports on triterpene saponins in several *Atriplex* species [24,25,26,28,32,33,34], in the current study we decided to investigate their presence in hitherto unexplored *A. sagittata*. As the extract of the flowers was the most active against hyaluronidase, we focused on its phytochemical analysis for the presence of saponins.

### 2.5. Isolation of Saponins from A. sagittata Flower Extract

**Figure 1:**
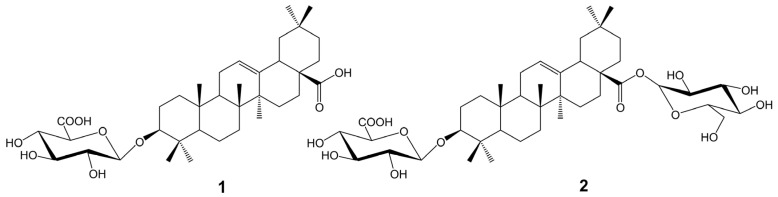
The structures of compound **1** and **2**.

Preliminary TLC of the methanolic extract of *A. sagittata* flowers revealed the presence of saponins. Therefore, this extract was fractionated by a combination of chromatographic methods (MPLC, CC) with the use of normal and reverse phase (RP-18). As a result, two compounds were isolated with a purity of more than 95%, as confirmed by liquid chromatography (LC-PDA). Their structures (Figure 1) were elucidated by analysis of their hydrolysis products, spectral data (NMR, MS) and comparison of the data obtained with those of published papers [73,74,75]. Compound **1** was identified as oleanolic acid-3-O-β-D-glucuronopyranoside (calenduloside E), while compound 2 was identified as 3-O-β-D-glucuronopyranosyl oleanolic acid 28-O-β-D-glucopyranosyl ester (chikusetsusaponin IVa). Calenduloside E (compound **1**) was previously isolated from *A. nummularia* [32], but chikusetsusaponin IVa (compound **2**) has not been found so far in any species of the genus *Atriplex*, although it is a saponin found in some species of the *Amarathaceae* family [76]. However, our study is the first report on the isolation of triterpene saponins from *A. sagittata.*

### 2.6. Antihyaluronidase Activity of Saponins from A. sagittata

**Figure 2:**
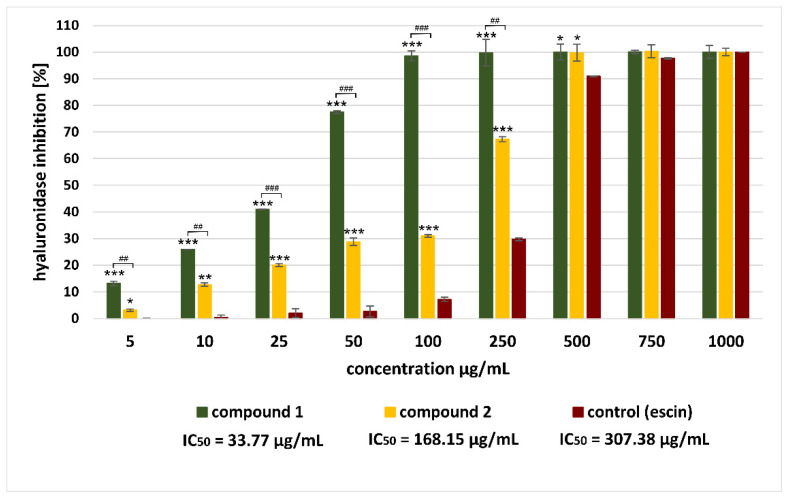
Antihyaluronidase activity of saponins (compound **1** and compound **2**) isolated from methanolic extract of *Atriplex sagittata* Borkh flowers. The results are presented as the mean± standard deviation (SD) calculated from six independent experiments. Values significantly different from the control (escin) are indicated by * for *p* < 0.05; ** *p* < 0.01, *** *p* < 0.001; Results for compound **1** significantly different vs. compound 2 are indicated by:, ^##^ *p* < 0.01, ^###^ *p* < 0.001.

In the next stage of the current investigation, the inhibitory effect of isolated saponins on the hyaluronidase enzyme was assessed. The results of the antihyaluronidase assay are shown in Figure 2.

The study revealed that both saponins from *A. sagittata* were potent enzyme inhibitors and acted in a dose-dependent manner (Figure 2). It should be noted that the observed activity was much higher than the effect demonstrated by a reference hyaluronidase inhibitor: escin (IC_50_ = 307.38 μg/mL) or quercetin (IC_50_ = 514.28 μg/mL). Among the saponins tested, compound **1** (calenduloside E) proved to be the strongest hyaluronidase inhibitor (IC_50_ = 33.77 μg/mL), while saponin **2** (chikusetsusaponin IVa) showed a weaker effect (IC_50_ = 168.15 μg/mL). Taking into account the structures of isolated compounds, our research is consistent with published reports indicating that the inhibitory effect on hyaluronidase may be related to the presence of a 3-*O*-*β*-D-glucuronopyranoside moiety in the sugar part of a saponin [77,78]. Furthermore, our study of these two compounds, which differ from each other only by the presence of a sugar moiety in the carbonyl group (28-COOH), also suggests the importance of the free carboxyl group in C-17 (28-COOH) in the oleanolic acid skeleton. We observed that the presence of a sugar moiety in the carbonyl group (28-COOH) significantly reduced the antihyaluronidase potential of compound 2 compared to compound **1**.

The high activity of isolated saponins, especially prominent in the case of compound **1**, suggests that they can significantly affect the overall antihyaluronidase activity of *A. sagittata* flower extract.

## 3. Materials and Methods

### 3.1. Chemicals and Reagents

Methanol and ethyl acetate were obtained from CHEMPUR (Gliwice, Poland), hydrochloric acid was obtained from STANLAB (Lublin, Poland). Phenolic acid standards: protocatechuic, gentisic, 4-hydroxybenzoic, vanillic, caffeic, syryngic, *p*-coumaric, ferulic, salicylic, sinapinic, gallic, rosmarinic, veratric, and 3-OH-cinnamic acid were obtained from Sigma-Aldrich Fine Chemicals (St. Louis, MO, USA). Methanol for LC was from J.T Baker (Phillipsburg, NJ, USA). Formic acid was purchased from Sigma-Aldrich Fine Chemicals (St. Louis, MO, USA). The standards of kaempferol-3-*O*-rutinoside, kaempferol-3-glucoside-7-rhamnoside, narcissoside (isorhamnetin-3-*O*-rutinoside), isoquercetin (quercetin-3-*O*-glucoside), LC grade acetonitrile were from Sigma-Aldrich Fine Chemicals (St. Louis, MO, USA). Isorhamnetin-3-*O*-glucoside was purchased from ChromaDex (Irvine, CA, USA). Naringin (naringenine-7-*O*-rhamnosidoglucoside), astragalin (kaempferol-3-*O*-glucoside), rutin (quercetin-3-*O*-rutinoside), isovitexin (apigenin-6-*C*-glucoside), vitexin (apigenin-8-*C*-glucoside) were from Roth (Karlsruhe, Germany). LC grade water was prepared using a Milipore Direct-Q3 purification system (Bedford, MA, USA). Trolox (6-hydroxy-2,5,7,8,-tetramethyl-chroman-2-carboxylic acid); FeCl_3_⋅× 6H_2_O; Folin–Ciocalteu reagent; 1,1-diphenyl-2-picrylhydrazyl (DPPH) were from Sigma Chemical Co. (St. Louis, MO, USA). Hydrochloric acid and sodium carbonate were from Avantor Performance Materials Poland S.A. (Gliwice, Poland). DMSO, Albumin from bovine serum: fraction V ≥ 98% (A3294), Hyaluronidase from bovine testes type I-S, Streptococcus equi hyaluronic acid, cetyltrimethylammonium bromide (CTAB), were obtained from Sigma-Aldrich. Acetate buffer pH 4.5 was purchased from J.T. Baker Chemical Co. (Phillipsburg, NJ, USA).

### 3.2. Plant Material

*Atriplex sagittata* Borkh was collected during the flowering phase, in Cracow—Pleszow, Poland (50°3′58.089″ N, 20°6′14.594″ E). The species identity was confirmed by Dr. Wacław Bartoszek of the Jagiellonian University Institute of Botany, Cracow, Poland. The voucher specimen No. ATR.NI/2016 has been deposited in the Department of Pharmacognosy of the Jagiellonian University Medical College, Cracow, Poland. The collected plant material was separated into parts (roots-R, stems-S, leaves-L, and flowers-F), finely chopped, and dried.

### 3.3. Extraction

Accurately weighed 5.0 g samples of plant material (leaves, flowers, stem and roots) were extracted under reflux (IKA rv 05-st, IKA^®^ Poland sp. Z.o.o. Warsaw, Poland) in a water bath at a temperature of 70° (IKA hb4 basic, IKA^®^ Poland sp. Z.o.o. Warsaw, Poland). The plant material was extracted with 50 mL of methanol three times and each extraction lasted for three hours. The combined extracts were filtered and concentrated under reduced pressure to obtain a volume of 50 mL. The extracts were then transferred to a 50 mL volumetric flask. 5 mL of each extract was transferred to amber glass vials and stored in a freezer (−20 °C) for further analysis (antioxidant activity and total phenolic content assays). The rest of the extracts were evaporated to dryness and stored in a freezer (−20 °C) for further analysis. The samples were prepared in six replicates.

### 3.4. Hydrolysis

Hydrolysis was performed according to Pyrzynska and Biesaga [50] with slight modifications [79]. 150 mg of methanolic extracts from each part of the plant were heated under reflux in a boiling water bath with 10 mL of 1.2 M HCl for two hours, protected from light. Subsequently, the samples were filtered and the filtrates were extracted three times by shaking with 10 mL of ethyl acetate for three minutes with four minute intervals. Organic fractions were collected, evaporated in vacuo, and dried by compressed air to a constant mass. The samples were stored in a freezer (−20 °C).

### 3.5. Quantitative Determination of Phenolic Acids and Flavonoids

#### 3.5.1. Sample Preparation

Before analysis, extract and hydrolyzate samples were redissolved in 80% methanol to obtain stock solutions and filtered through a membrane filter with 0.45 μm membrane pores.

#### 3.5.2. LC-ESI-MS/MS Analysis

Phenolic acids and flavonoids were determined by reversed phase high-performance liquid chromatography and electrospray ionization mass spectrometry (LC-ESI-MS/MS). The Agilent 1200 Series HPLC system (Agilent Technologies, Santa Clara, CA, USA) was equipped with a binary gradient solvent pump, a degasser, an autosampler, and a column oven connected to a 3200 QTRAP mass spectrometer (AB Sciex, Framingham, MA, USA). The separation of phenolic acids was carried out at 25 °C on a Zorbax SB-C18 column (2.1 × 50 mm, 1.8-μm particle size; Agilent Technologies, Santa Clara, CA, USA). Gradient elution was applied using water containing 0.1% HCOOH (A) and methanol (B). The gradient was as follows: 0–0.8 min 5% B; 2–3 min 20% B; 5–7.5 min 100% B; 8.5–11 min 5% B. The injection volume was 3 µL and the flow rate was 400 µL/min).

In turn, the separation of flavonoid glycosides was achieved at 25 °C on an Eclipse XDB-C18 column (4.6 × 150 mm, 5-μm particle size; Agilent Technologies, Santa Clara, CA, USA), using water containing 0.1% HCOOH (solvent A) and acetonitrile with 0.1% HCOOH (solvent B). The gradient was as follows: 0–2 min 15% B; 4–5 min 25% B; 6–9 min 35% B; 11–16 min 60% B; 18–21 min 80% B. The total run time was 28 min. The injection volume was 3 µL and the flow rate was 400 µL/min.

Detector: A 3200 QTRAP mass spectrometer MS/MS system and an electrospray ion source in the negative mode were used. The conditions were as follows: for phenolic acids curtain gas 30 psi, capillary temperature 500 °C, for flavonoid glycosides curtain gas 23 psi, capillary temperature 450 °C. Both nebulizer gases were 60 psi, negative ionization mode source voltage −4500 V. Nitrogen was used as the nebulizer and collision gas. Data were acquired and processed using Analyst 1.5 software from AB Sciex, Framingham, MA, USA. The analytes were identified by comparing the retention times and *m*/*z* values obtained by MS and MS2 with the mass spectra of the corresponding standards tested under the same conditions. The calibration curves obtained in the MRM mode were used for quantification of all analytes. The limits of detection (LOD) and quantification (LOQ) were determined in a signal-to-noise ratio of 3:1 and 10:1, respectively, by injecting a series of dilute solutions of known concentrations. The summary of optimized parameters for quantitative analysis of flavonoid glycosides and phenolic acids is presented in Tables S1–S4 (Supplementary Materials).

All analyzes were performed in triplicate and the mean values of the individual compounds (flavonoids or phenolic acids) were expressed as µg/g of the sample (extract or hydrolyzate) and finally calculated and expressed as µg/g of dry plant material (dw.). Furthermore, based on the results obtained concerning the content of individual compounds in 1 g of dry plant material, the sum of identified flavonoids (Flav. SUM) and the sum of identified phenolic acids (PA SUM) present in 1 g of dry plant material were calculated, and this value is expressed as µg/g dw.

### 3.6. Isolation of Saponins

Preliminary TLC of methanolic extracts (silica gel, CHCl_3_-CH_3_OH-H_2_O (23:12:2 *v*/*v*), 25% H_2_SO_4_ in CH_3_OH + heating) of flowers suggested the presence of saponins in flowers. To isolate saponins, extract from a larger sample of flowers (300 g) was prepared in an analogous manner as described above (Section 3.3). The MeOH extract was evaporated under reduced pressure on a rotary evaporator to dryness to yield 15 g (extraction yield 5%) of greenish residue, which was then suspended in water and extracted with n-BuOH. Then, the n-BuOH extract was concentrated in vacuo to yield 6 g of dark brown residue. The portions of the n-BuOH extract (2 g) were fractionated by medium pressure chromatography (MPLC; Sepacore apparatus; BÜCHI Labortechnik AG, Flawil, Switzerland) on silica gel under conditions previously described [80] to give eight fractions (A1–A8). The fraction A4 was further fractionated by column chromatography (CC, column 12 × 450 mm; silica gel) using the following isocratic solvent system: CHCl_3_-CH_3_OH-H_2_O (20:12:2 *v*/*v*). The fractions were combined on the basis of TLC (silica gel, CHCl_3_-CH_3_OH-H_2_O (20:12:2 *v*/*v*), 25% H_2_SO_4_ in CH_3_OH + heating) to give seven fractions (B1–B7). Fraction B4 was purified by MPLC (MPLC column 12 × 150 mm; flow rate: 2.6 mL/min) on reverse phase silica gel (LiChroprep, RP-18 (40–63 μm); Merck, Darmstadt, Germany) using an isocratic solvent system (CH_3_OH-H_2_O (7:1.5)). The fractions were combined by TLC examination (silica gel 60 plates, Merck, developed with the solvent system CH_3_OH-H_2_O (7:1.5 *v*/*v*). Chromatograms were visualized by spraying TLC plates (RP-18; silica gel 60 RP-18 F_254_S; Supelco, Mainz, Germany) with 25% H_2_SO_4_ in MeOH, followed by heating. The fractionation of fraction B4 led to the isolation of compound **1** (23 mg). The fraction A6 was chromatographed by column chromatography (CC, column 15 × 40 mm) using the following solvent system: CHCl_3_-CH_3_OH-H_2_O (30:20:4 *v*/*v*) to give six fractions (C1-C6). The C3 fraction was further purified by MPLC on a reverse phase silica gel column (LiChroprep, RP-18 (40–63 μm); Merck, Darmstadt, Germany) column (MPLC column 12 × 150 mm; flow rate: 2.9 mL/min) using an isocratic solvent system (CH_3_OH-H_2_O (7:3)). The eluates were controlled by TLC examination (RP-18; silica gel 60 RP-18 F_254_S; Supelco, Mainz, Germany; CH_3_OH-H_2_O (7:3); 25% H_2_SO_4_ in MeOH + heating) The separation process gave 26 mg of compound **2**.

### 3.7. Structure Elucidation of Isolated Compounds

The acid hydrolysis of isolated compounds (**1** and **2**) was carried out using the method described previously: sugar analysis [81]; sapogenin analysis [77]. NMR spectra were recorded in pyridine-d5 on the JNM-ECZR500 RS1 500 MHz (JEOL), using a standard pulse sequence at 500 MHz. LC-MS analysis was performed on a UPLC/MS Waters ACQUITY TQD (Waters Corporation, Milford, MA, USA) on the Acquity UPLC BEH (bridged ethyl hybrid) C18 column (2.1 × 100 mm, and particle size of 1.7 μm), equipped with the Acquity UPLC BEH C18 VanGuard precolumn (2.1 × 5 mm, and particle size of 1.7 μm) using conditions described previously [80]. Chromatograms were recorded using the Waters eλ PDA detector. Spectra are provided in the Supplementary Material (Figures S2–S9).

#### 3.7.1. Compound **1**: Oleanolic acid-3-O-β-D-glucuronopyranoside (Calenduloside E) (Figure S1)

White powder; ^1^H and ^13^C NMR: see Table S5, Figures S4 and S5 (Supplementary Materials).

ESI (positive ion mode) *m*/*z* 633.51 [M + H]^+^, fragmentation in MS/MS: *m*/*z* 439.31 [M-H-176–18]^+^; ESI MS (negative ion mode) *m*/*z* 631.19 [M-H]^−^ (Figure S3).

#### 3.7.2. Compound **2**: 3-O-β-D-glucuronopyranosyl Oleanolic acid 28-O-β-D-glucopyranosyl Ester (Chikusetsusaponin IVa) (Figure S1)

White powder; ^1^H and ^13^C NMR: see Table S5, Figures S8 and S9 (Supplementary Materials).

ESI MS (negative ion mode) *m*/*z* 793.36 [M-H]^−^; ESI (positive ion mode)—fragmentation: *m*/*z* 439.31 [M-H-162-176-18]^+^ (Figure S7).

### 3.8. Determination of the Total Phenolic Content (TPC)

Total phenolics (TP) were colorimetrically determined using Folin–Ciocalteau reagent, as previously described [82]. The absorption of the mixture was measured at 725 nm. A standard curve was prepared with gallic acid. The final results were given as mg GAE/100 g of dw.

### 3.9. Determination of Antioxidant Activity

The analysis was performed using the DPPH and FRAP methods. The FRAP assay was previously described and modified to 48-well plates and BioTek Synergy multiplate reader with syringe rapid dispensers. Briefly, the reagent mixture consisting of ferric chloride solution (20 mmol/L), TPTZ solution (10 mmol/L TPTZ in 40 mmol/L HCl) and acetate buffer (pH = 3.6) in the proportion of 5:5:10, respectively, was freshly prepared. To each plate, 0.4 mL of acetate buffer was added, followed by 50 µL of sample, blank or standard. The plate was conditioned at a temperature of 37 °C for 2 min, and then 0.2 mL of the previously described reagent mixture was added and shaken for 30 s; afterwards, absorbance at 593 nm was measured in kinetic mode for 8 min. [64,82]. The final results were expressed as mmol Fe^2+/^100 g dw. DPPH radical-scavenging activity was measured according to a scheme similar to that described earlier by Barton [83]. Briefly, in 12 rows of a 96-well microplate, the following reagents were injected: 50 μL of 1M acetate buffer in methanol, increasing amounts of sample in ethanol (0–16 μL), than decreasing amounts of ethanol to make the combined sample volume of 16 μL, and 34 μL of methanol; then the plate was thermostated at 25 °C and finally 100 μL of 0.3 mM DPPH radical in methanol was added. The plate was covered with a transparent lid, sealed with parafilm, thermostated at 25 °C, and scanned at 515 nm for 1 h at minute intervals. The residual DPPH was decolorized by injection of concentrated Trolox solution in methanol (20 μL of Trolox solution 1.5 mg/mL in methanol), then the absorbance was read at 515 nm. The spectral sample background of the initial mixture was evaluated after measurement, and background correction was performed by a simplified one-step correction. Scavenging efficacy was calculated as a percentage of decolorization based on the corrected absorbance. Standard Trolox equivalent antioxidant capacity at zero sample concentration (TEAC_0_) was obtained by extrapolating to zero sample concentration by linear regression, as previously described [83]. The final results were expressed as mmol Trolox/100 g dw.

### 3.10. Determination of Antihyaluronidase Activity

Antihyaluronidase activity was determined using a spectrophotometric method described previously [77]. Briefly, extracts after evaporation of methanol were dissolved in DMSO (at concentrations of 0.01–1 mg/mL) and were pre-incubated (10 min, 37 °C) in the presence of the hyaluronidase enzyme (25 µL Hyal, 30 U/mL) and incubation buffer (25 µL, acetate buffer, pH 4.5, 77 nM NaCl, 0.5 mg/mL albumin). The same procedure was applied to isolated compounds (at concentrations 0.005–1 mg/mL). Next, a solution of hyaluronic acid (25 µL HA, 0.3 mg/mL) was added to the reaction mixtures and the samples were incubated (45 min, 37 °C). After incubation, CTAB (200 µL of 2.5%) solution was added to the reaction mixtures. The inhibitory effect of the tested extracts and saponins on the enzyme activity was determined on the basis of the measurement of the absorbance of the precipitated nonhydrolyzed hyaluronic acid. The study was carried out using a multipurpose plate reader (Synergy HT BioTek, Winooski, VT, USA) at a wavelength of 600 nm. Antihyaluronidase activity was expressed as % enzyme inhibition, as previously described [77]. Quercetin and escin were used as positive controls in the range of concentrations (10–1000 µg/mL corresponding to the analyzed substances (see Table 4).

### 3.11. Statistical Analysis

Data were analyzed using Statistica v.13.3 (StatSoft, Tulsa, OK, USA). The results were expressed as mean (±SD). The statistical significance between the samples in the quantification study was determined using analysis of variance (Welch’s ANOVA) and the post hoc Tukey multiple comparison test. One-way analysis of variance (ANOVA) and the post hoc Tukey multiple comparison test were used to check the differences between extracts in the antihyaluronidase study. One-way analysis of variance (ANOVA) and the post hoc Duncan test were used to check differences between extracts in the antioxidant activity study. The probability level of *p* < 0.05 was considered statistically significant.

## 4. Conclusions

The results obtained in the current study suggest that *Atriplex sagittata* Borkh, like other *Atriplex* species, produces high amounts of flavonoids and phenolic acids, among other compounds with high biological activity, which play an important role in natural medicine and plant physiology.

Soluble phenolic acids are present in all plant parts of *A. sagittata*, in free and conjugated form, however, the latter form predominates. The highest content of phenolic acids (free and conjugated) and flavonoids was found in leaves and flowers. The most common phenolics were 4-hydroxybenzoic and salicylic acids, kaempferol-3-glucoside-7-rhamnoside, kaempferol-3-rutinoside and the rare narcissoside are present in almost all morphotic parts. In turn, gentisic acid, kaempferol-3-rutinoside, kaempferol-3-glucoside-7-rhamnoside, and apigenin derivatives were detected in *Atriplex* species for the first time. Quantitative determination of soluble conjugated phenolic acids was also performed for the first time in any of the *Atriplex* species. However, the detection of these forms indicates the need for further research that will allow determination of the full structure of these compounds and their biological activity. *A. sagittata* is a plant species commonly distributed worldwide. It should be emphasized that the conducted analyzes concern plant material from central Europe. Further research should investigate whether geographical location influences differences in the quantitative and qualitative profile of these compounds.

The extracts from different morphotic parts of *A. sagittata* exhibited various antioxidant effects. The results of the current study indicate that not only identified and quantified phenolic compounds may be responsible for this activity. In addition, the observed potential may be the result of the synergy of the compounds.

This is also the first report on the antihyaluronidase activity of extracts from *Atriplex* species, and the results show that all the extracts analyzed exhibited a potent effect, higher than the reference substances (quercetin and escin). The flower extract turned out to be the most active. Its phytochemical analysis led to the isolation of two saponins: oleanolic acid-3-*O*-*β*-D-glucuronopyranoside (calenduloside E) and 3-*O*-*β*-D-glucuronopyranosyl oleanolic acid 28-*O-β*-D-glucopyranosyl ester (chikusetsusaponin IVa), with a strong inhibitory potential of hyaluronidase. This is the first report on the presence of saponins in *A. sagittata*.

The current study suggests that not only phenolic compounds, but also saponins may affect the biological activity of extracts. Furthermore, *A. sagittata* should be considered as a potential source of compounds helpful in diseases related to excessive hyaluronidase activity and loss of hyaluronic acid, e.g., osteoarthritis, and also cosmetology, in cases of premature aging of the skin corresponding to a decrease in the level of hyaluronic acid. However, further experiments corresponding to in vivo conditions are required to confirm these in vitro observations. It should be emphasized that the use of class 2 solvents, such as methanol, as extractants in future in vivo studies entails consideration of safety standards. The potential manufacture of plant products containing dry methanol extracts requires compliance with the ICH standards [84].

## Figures and Tables

**Table 1:** Phenolic acid content in different morphological parts of *Atriplex sagittata* Borkh. before and after acidic hydrolysis [μg/g dw. ±SD].

Compound	Sample							
	Flower		Leaves		Stem		Root	
	Extract	Hydrolyzed Extr.	Extract	Hydrolyzed Extr.	Extract	Hydrolyzed Extr.	Extract	Hydrolyzed Extr.
ferulic acid	18.57 ± 3.05 ^a^	2.99 ± 1.23	22.59 ± 1.16 ^a^	36.53 ± 7.27	<LOD	<LOQ	<LOD	<LOQ
protocatechuic acid	<LOQ	26.04 ± 3.32	Nd	14.13 ± 2.21	<LOQ	<LOQ	<LOD	<LOQ
gentisic acid	<LOQ	2.05 ± 0.76	Nd	26.59 ± 5.57	<LOQ	<LOQ	Nd	0.64 ± 0.21
4-hydroxybenzoic acid	12.80 ± 4.42	52.12 ± 7.66	21.25 ± 1.30	84.29 ± 5.84	<LOQ	1.46 ± 0.82	<LOQ	4.03 ± 1.16
salicylic acid	13.83 ± 2.93 ^a^	11.07 ± 1.9 ^a^	<LOQ	1.82 ± 0.62	<LOQ	0.27 ± 0.10	<LOQ	0.59 ± 0.15
4-hydroxycynamic acid	<LOQ	<LOQ	<LOQ	3.28 ± 1.19	<LOQ	<LOQ	<LOQ	<LOQ
vanillic acid	Nd	21.26 ± 7.21 ^a^	Nd	20.25 ± 6.24 ^a^	Nd	<LOQ	Nd	4.33 ± 0.65
caffeic acid	Nd	Nd	Nd	1.25 ± 0.23	Nd	Nd	Nd	Nd
syringic acid	<LOQ	<LOQ	<LOQ	33.26 ± 9.03	<LOQ	5.58 ± 2.39 ^a^	<LOQ	7.10 ± 2.60 ^a^
sinapic acid	Nd	Nd	Nd	3.84 ± 2.04	<LOQ	<LOQ	Nd	<LOQ
3-hydroxycynamic acid	<LOQ	<LOQ	<LOQ	Nd	Nd	Nd	Nd	Nd
Other ^#^	Nd	Nd	Nd	Nd	Nd	Nd	Nd	Nd
Sum *	45.20 ± 10.4 ^a^	115.53 ± 22.15	43.84 ± 2.46 ^a^	225.24 ± 40.29	0.00	6.45 ± 3.32	0.00	16.68 ± 4.78

The results are presented as the mean± standard deviation (SD) calculated from six independent experiments. Abbreviations: ^#^ other analyzed phenolic acids: rosmarinic, gallic, veratric; <LOQ—peak detected but concentration lower than limit of quantitation (LOQ); *—the sum of quantified compounds—the value calculated on the basis of the content of individual components in 1 g of dried plant material. The results marked with the letter ^a^ within each row did not differ significantly (*p* > 0.05). Results not marked with the letter within each row differ significantly from others (*p* < 0.05).

**Table 2:** Flavonoid contents in different morphological parts of *Atriplex sagittata* Borkh. [μg/ g dw. ±SD].

Compound	Plant Part			
	Flowers	Leaves	Stem	Root
Astragalin	<LOQ	77.38 ± 2.25	<LOQ	<LOQ
Kaempferol-3-rutinoside	4.97 ± 0.12	9.54 ± 0.40	0.24 ± 0.01	<LOQ
Kaempferol-3-glucoside-7-Rhamnoside	73.76 ± 2.72	97.13 ± 2.82	8.61 ± 0.37	<LOQ
Vitexin/isovitexin	29.55 ± 0.75	Nd	Nd	Nd
Rutin	<LOQ	2.82 ± 0.09	<LOQ	<LOQ
Isoquercetin	100.84 ± 2.75	<LOQ	<LOQ	<LOQ
Narcisoside	33.59 ± 0.57	15.99 ± 0.03	4.19 ± 0.79	Nd
Isorhamnetin-3-glucoside	<LOQ	<LOQ	<LOQ	<LOQ
Naringin	<LOQ	Nd	Nd	Nd
Other flavonoids ^#^	Nd	Nd	Nd	Nd
Sum *	242.71 ± 6.91	202.86 ± 5.59	13.05 ± 1.17	0.00

The results are presented as the mean± standard deviation (SD) calculated from six independent experiments. Abbreviations: ^#^ Other Flav—apigenin-7-glucoside, quercetin-7-glucoside, hyperoside, tiliroside, naringenin-7-glucoside, luteolin 7-glucoside, kaempferol-3,7-diramnoside, narirutin, eriocitrin, robinin, quercetin-3,7-diramnoside, eleuteroside E, kaempferol-4′-rutinoside, luteolin-3,7-diglucoside, eriodictyol-7-glucopyranoside; <LOQ—peak detected but concentration lower than limit of quantitation (LOQ); Nd—below limit of detection (LOD); *—sum of quantified compounds—the value calculated on the basis of the content of individual components in 1 g of dry plant material. Results marked with the same letter within each row did not differ significantly (*p* > 0.05). Results not marked with the letter within each row differ significantly from others (*p* < 0.05).

**Table 3:** Total phenolic content (TPC), sum of flavonoids and phenolic acids, and antioxidant activity of methanolic extracts from different morphological parts of *Atriplex sagittata* Borkh.

Plant Part	TPC [mg GAE/100 g dw.]	Flav. SUM [μg/g dw.]	PA SUM [μg/g dw.]	Antioxidant Potential	
				FRAP[mmolFe^2+^/ 100 g dw.]	DPP HmmolTrolox/100 g dw.)
Flowers	85.36 ± 3.00	242.71 ± 6.91	45.20 ± 10.4	0.44 ± 0.05 ^a^	0.16 ± 0.009 ^a^
Leaves	169.91 ± 1.4	202.86 ± 5.59	43.84 ± 2.46	0.70 ± 0.03 ^b^	0.32 ± 0.04
Stem	611.86 ± 10.42	13.05 ± 1.17	0.00	5.46 ± 0.21	2.99 ± 0.26
Root	59.16 ± 1.16	0.00	0.00	0.59 ± 0.04 ^a,b^	0.13 ± 0.02 ^a^

The results are presented as the mean ± standard deviation (SD). The results marked with the same letter within each column did not differ significantly (*p* > 0.05). Results not marked with the letter within each column differ significantly from others (*p* < 0.05). Abbreviations: Flav. SUM = sum of quantified individual flavonoids; PA SUM = sum of quantified individual phenolic acids; Flav. SUM and PA SUM—values calculated on the basis of the content of individual components in 1 g of dry plant material; TPC = total phenolic content determined colorimetrically.

**Table 4:** Antihyaluronidase activity of extracts of different morphological parts of *Atriplex sagittata* Borkh.

	Hyaluronidase Inhibition [%]				
Concentration [µg/mL]	Control	Flowers	Leaves	Stem	Root
1000	91.11 ± 1.50	100.00 ± 0.01 ^a^	100.00 ± 0.001 ^a^	100.00 ± 0.001 ^a^	99.79 ± 0.36 ^a^
700	80.56 ± 0.49	100.00 ± 0.01 ^a^	99.12 ± 0.38 ^a^	97.34 ± 0.57 ^a^	98.11 ± 0.63 ^a^
500	38.84 ± 1.33	98.33 ± 1.89 ^a^	93.90 ± 1.37 ^b^	96.83 ± 0.54 ^a,b^	87.16 ± 2.18
300	21.86 ± 4.04	95.93 ± 1.70	87.47 ± 0.74	66.67 ± 0.34	59.38 ± 1.88
200	13.86 ± 2.70	94.45 ± 2.19	37.39 ± 1.96	30.88 ± 2.48	24.05 ± 0.63
100	4.39 ± 0.88	61.61 ± 4.67	13.20 ± 2.28 ^a^	11.31 ± 1.19 ^a^	6.94 ± 0.41
50	1.24 ± 0.99	17.72 ± 1.52	5.29 ± 1.21 ^a^	5.26 ± 1.39 ^a^	NA
20	0.62 ± 0.54 ^a^	0.76 ± 0.32 ^a^	NA	NA	NA
10	0.61 ± 0.53	NA	NA	NA	NA
0	NA	NA	NA	NA	NA
**IC_50_**	**514.28**	**84.67**	**216.2**	**244.5**	**272.5**

The results are presented as the mean ± standard deviation (SD) calculated from six independent experiments. Concentration refers to dry methanolic extract dissolved in DMSO; Control = quercetin; NA—not active; Results marked with the same letter (^a,b^) within each row did not differ significantly (*p* > 0.05). Results not marked with the letter within each row differ significantly from others (*p* < 0.05).

## Data Availability

Not applicable.

## Supplementary Materials

The following supporting information can be downloaded at: https://www.mdpi.com/article/10.3390/molecules28030982/s1, Table S1. Optimized parameters for the quantitative analysis of phenolic acids. Table S2. Analytical parameters of the quantitative LC-MS/MS method for determination of phenolic acids. Table S3. Optimized parameters for the quantitative analysis of flavonoids. Table S4. Analytical parameters of the quantitative LC-MS/MS method for determination of flavonoids. Figure S1. Structures of compounds **1** (calenduloside E) and **2** (chikusetsusaponin IVa). Figure S2. UPLC-PDA chromatogram of compound **1**. Figure S3 ESI-QTOF-MS (negative and positive ion mode) of compound **1**. Figure S4. ^1^H NMR (500 MHz, pyridine-d5) spectrum of compound **1**. Figure S5. ^13^C NMR (125 MHz, pyridine-d5) spectrum of compound **1**. Figure S6. UPLC-PDA chromatogram of compound **2.** Figure S7. ESI-QTOF-MS (negative and positive ion mode) of compound **2**. Figure S8. ^1^H NMR (500 MHz, pyridine-d5) spectrum of compound **2**. Figure S9. ^13^C NMR (125 MHz, pyridine-d5) spectrum of compound **2**. Table S5. ^1^H (500 MHz) and ^13^C (125 MHz) NMR spectral data (δ ppm) for compound **1** and **2** (pyridine-d5).

## Sample Availability

Samples of the compounds are not available from the authors.
