# Supplementary material for: Antihyaluronidase and Antioxidant Potential of Atriplex sagittata Borkh. in Relation to Phenolic Compounds and Triterpene Saponins
Source: Molecules. 2023 Jan 18;28(3):982. doi: 10.3390/molecules28030982 (PMC9921161; doi:10.3390/molecules28030982)
Supplement: Supplementary file 1 [file molecules-28-00982-s001.zip › molecules-2079923-supplementary.pdf]

Supplementary Materials

Antihyaluronidase and Antioxidant Potential of *Atriplex sagittata* Borkh. in Relation to Phenolic Compounds and Triterpene Saponins

Karolina Grabowska [1], Wioleta Pietrzak [2], Paweł Paśko [3], Agnieszka Sołtys [1], Agnieszka Galanty [1], Paweł Żmudzki [4], Renata Nowak [2] and Irma Podolak [1,*]

[1] Department of Pharmacognosy, Medical College, Jagiellonian University, Medyczna 9, 30-688 Kraków, Poland
[2] Department of Pharmaceutical Botany, Medical University, 1 W. Chodźki, 20-093 Lublin, Poland
[3] Department of Food Chemistry and Nutrition, Medical College, Jagiellonian University, 9 Medyczna, 30-688 Kraków, Poland
[4] Department of Medicinal Chemistry, Medical College, Jagiellonian University, 9 Medyczna, 30-688 Kraków, Poland
* Correspondence: irma.podolak@uj.edu.pl; Tel.:+48-(12)-620-55-60

Table of contents:

Table S1. Optimized parameters for the quantitative analysis of phenolic acids.
Table S2. Analytical parameters of the quantitative LC-MS/MS method for determination of phenolic acids.
Table S3. Optimized parameters for the quantitative analysis of flavonoids.
Table S4. Analytical parameters of the quantitative LC-MS/MS method for determination of flavonoids.
Figure S1. Structures of compounds 1 (calenduloside E) and 2 (chikusetsusaponin IVa).
Figure S2. UPLC-PDA chromatogram of compound 1.
Figure S3 ESI-QTOF-MS (negative and positive ion mode) of compound 1.
Figure S4. ^{1}H NMR (500 MHz, pyridine-d5) spectrum of compound 1.
Figure S5. ^{13}C NMR (125 MHz, pyridine-d5) spectrum of compound 1.
Figure S6. UPLC-PDA chromatogram of compound 2.
Figure S7. ESI-QTOF-MS (negative and positive ion mode) of compound 2.
Figure S8. ^{1}H NMR (500 MHz, pyridine-d5) spectrum of compound 2.
Figure S9. ^{13}C NMR (125 MHz, pyridine-d5) spectrum of compound 2.
Table S5. ^{1}H (500 MHz) and ^{13}C (125 MHz) NMR spectral data (δ ppm) for compound **1** and **2** (pyridine-d5).

Table S1. Optimized parameters for the quantitative analysis of phenolic acids.

Compound	Retention time [min]	MRM (Q1/Q3) [m/z]	DP [V]	EP [V]	CEP [V]	CE [eV]	CXP [V]
gallic acid	1.01	168.7/78.9	-35	-3	-12	-36	0
		168.7/124.9	-35	-3	-12	-14	0
protocatechuic	2.25	152.9/80.9	-55	-1	-10	-26	0
		152.9/107.8	-55	-1	-10	-38	0
gentisic acid	3.34	352.9/80	-70	-4	-16	-110	0
		352.9/96.9	-70	-4	-16	-52	0
4-hydroxybenzoic acid	4.24	136.8/92.9	-30	-7	-10	-18	0
vanillic acid	5.52	166.8//107.9	-35	-4	-12	-18	0
		166.8/123	-35	-4	-12	-12	0
caffeic acid	5.75	178.7/88.9	-30	-6,5	-12	-46	0
		178.7/134.9	-30	-6,5	-12	-16	0
syringic acid	6.34	196.9/122.8	-30	-9	-12	-24	0
		196.9/181.9	-30	-9	-12	-12	-2
4-hydroxycynamic acid	6.70	162.8/93	-30	-8	-12	-44	0
		162.8/119	-30	-8	-12	-14	0
ferulic acid	6.84	192.8/133.9	-25	-11,5	-14	-16	0
		192.8/177.9	-25	-11,5	-14	-12	-2
salicylic acid	6.86	136.9/75	-35	-4	-10	-48	0
		136.9/93	-35	-4	-10	-16	-2
sinapic acid	6.87	222.8/121	-35	-8,5	-10	-36	0
		222.8/148.9	-35	-8,5	-10	-20	0
veratric acid	6.88	180.7/121.9	-35	-6	-14	-18	0
		180.7/136.9	-35	-6	-14	-12	0
3-hydroxycynamic acid	6.89	162.8/91	-35	-4,5	-12	-36	0
		162.8/119	-35	-4,5	-12	-14	0
rosmarinic acid	7.01	358.7/132.6	-50	-5	-26	-44	0
		358.7/160.8	-50	-5	-26	-20	-2

Abbreviations: Q1 – precursor ion, Q3 – product ion, [a]DP- Declustering Potential; - [b]EP- Entrance Potential; [c]CEP- Cell Entrance Potential; [d]CE- Collision Energy; [e]CXP- Collision Cell Exit Potential.

Table S2. Analytical parameters of the quantitative LC-MS/MS method for determination of phenolic acids.

Compound	LOD [ng/mL]	LOQ [ng/mL]	R^2	Linear range [ng/mL]
gallic acid	50	100	0.9995	100-50000
protocatechuic	10	60	0.9993	60-12000
gentisic acid	8	15	0.9982	30-7500
4-hydroxybenzoic acid	30	50	0.9990	50-50000
vanillic acid	60	120	0.9994	120-30000
caffeic acid	60	315	0.9976	313-6250
syringic acid	50	155	0.9986	155-7750
4-hydroxycynamic acid	20	30	0.9980	30-7500
ferulic acid	15	25	0.9982	25-5000
salicylic acid	5	10	0.9979	44-1000
sinapic acid	7	30	0.9992	30-6000
veratric acid	100	500	0.9975	500-25000
3-hydroxycynamic acid	25	100	0.9994	100-25000
rosmarinic acid	20	25	0.9995	25-25000

Abbreviations: LOD -limit of detection. LOQ -limit of quantification. R^2 - determination coefficient.

Table S3. Optimized parameters for the quantitative analysis of flavonoids.

Compound	Retention time [min]	Q1 [m/z]	Q3 [m/z]	DP[a] [V]	EP[b] [V]	CEP[c] [V]	CE[d] [eV]	CXP[e] [V]
kaempferol-3-glucoside-7-rhamnoside	11.25	592.818	284.9	-145	-4.5	-30	-42	-4
			430.9	-145	-4.5	-30	-36	-16
rutin	11.99	608.745	299.6	-90	-8	-30	-46	-4
			270.9	-90	-8	-30	-60	-4
isovitexin/vitexin	12.4	430.821	310.9	-65	-4.5	-18	-28	-4
			340.9	-65	-4.5	-18	-26	-14
isoquercetin	13	462.739	299.7	-85	-1.5	-20	-30	-4
			270.7	-85	-1.5	-20	-44	-4
kaempferol-3-rutinoside	13.31	592.697	284.8	-65	-12	-30	-38	-2
			226.7	-65	-12	-30	-68	-2
narcisoside	13.52	622.817	314.9	-90	-4.5	-30	-40	-4
			298.8	-90	-4.5	-30	-52	-4
naringin	14.50	579.109	151	-80	-4	-26	-54	-2
			271	-80	-4	-26	-42	-4
astragalin	14.66	446.72	226.8	-75	-9	-20	-54	-2
			254.8	-75	-9	-20	-40	-2
isorhamnetin-3-glucoside	14.76	476.807	313.9	-95	-10	-22	-30	-4
			270.9	-95	-10	-22	-44	-4

Abbreviations: Abbreviations: Q1 – precursor ion, Q3 – product ion [a] DP- Declustering Potential; - [b] EP- Entrance Potential; [c] CEP- Cell Entrance Potential; [d] CE- Collision Energy; [e] CXP- Collision Cell Exit Potential.

Table S4. Analytical parameters of the quantitative LC-MS/MS method for determination of flavonoids.

Compound	LOD [ng/mL]	LOQ [ng/ mL]	R^2	Linear range [ng/ mL]
kaempferol-3-glucoside-7-rhamnoside	90	150	0.9980	400-40000
rutin	100	250	0.9973	2000-28000
isovitexin/vitexin	100	250	0.9989	2500-80000
isoquercetin	167	250	0.9987	2000-25000
kaempferol-3-rutinoside	50	100	0.9991	200-20000
narcisoside	50	100	0.9975	200-20000
naringin	50	100	0.9985	1000-25000
astragalin	120	240	0.9978	1200-24000
isorhamnetin-3-glucoside	100	200	0.9985	2000-20000

Abbreviations: LOD -limit of detection. LOQ -limit of quantification. R^2 - determination coefficient.

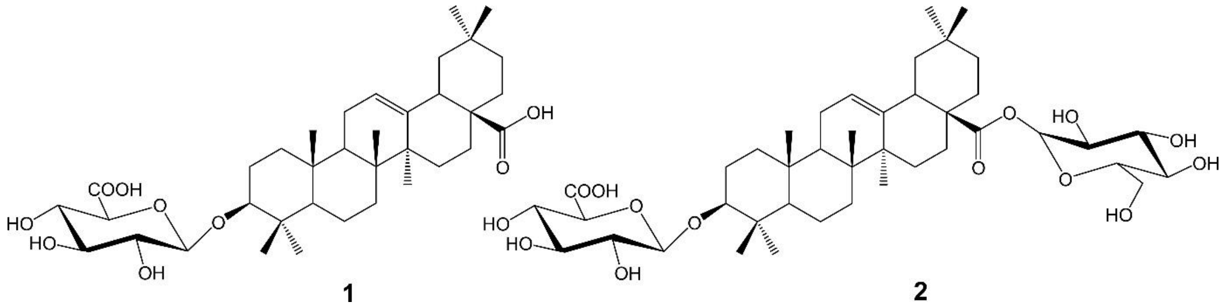

Figure S1. Structures of compounds 1 (calenduloside E) and 2 (chikusetsusaponin IVa).

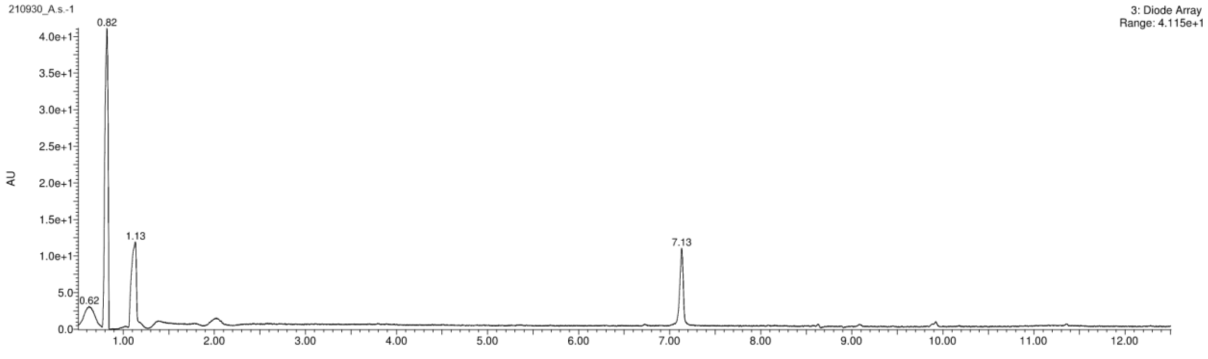

Figure S2. UPLC-PDA chromatogram of compound 1.

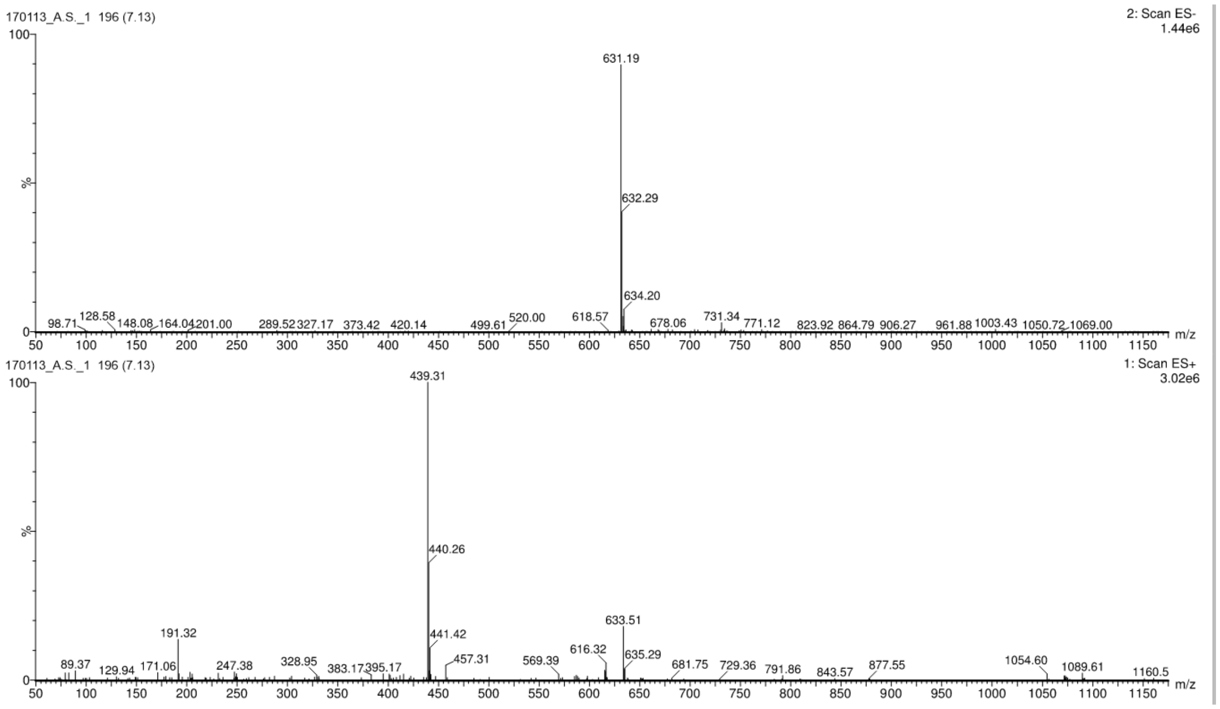

Figure S3. ESI-QTOF-MS (negative and positive ion mode) of compound 1.

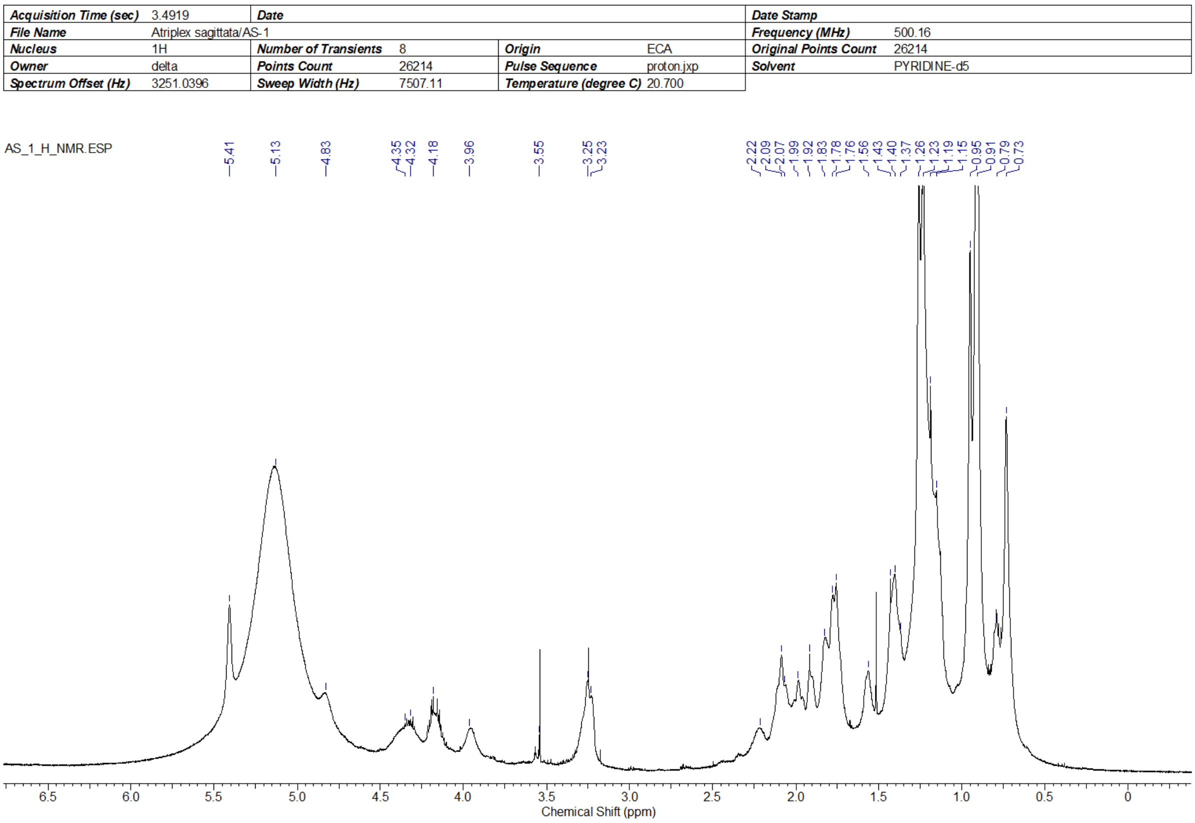

Figure S4. The ^{1}H NMR (500 MHz, pyridine-d5) spectrum of compound 1.

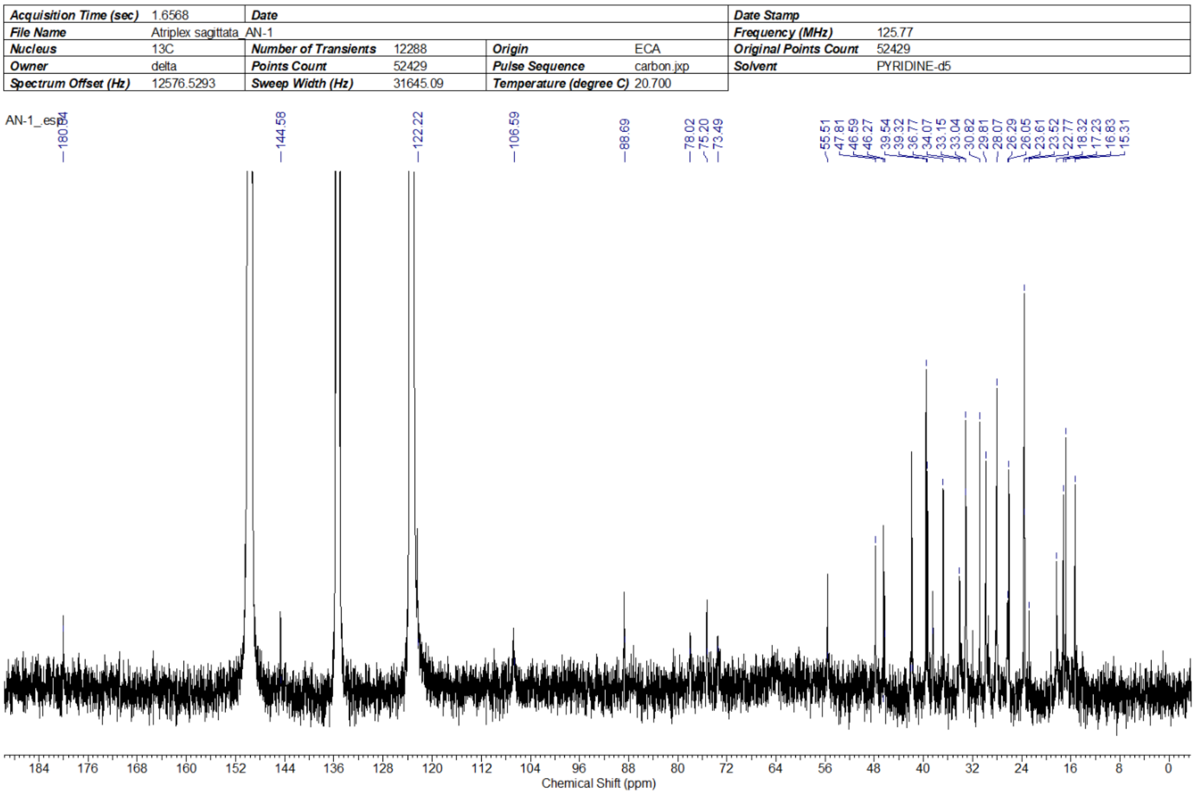

Figure S5. The ^{13}C NMR (125 MHz, pyridine-d5) spectrum of compound 1.

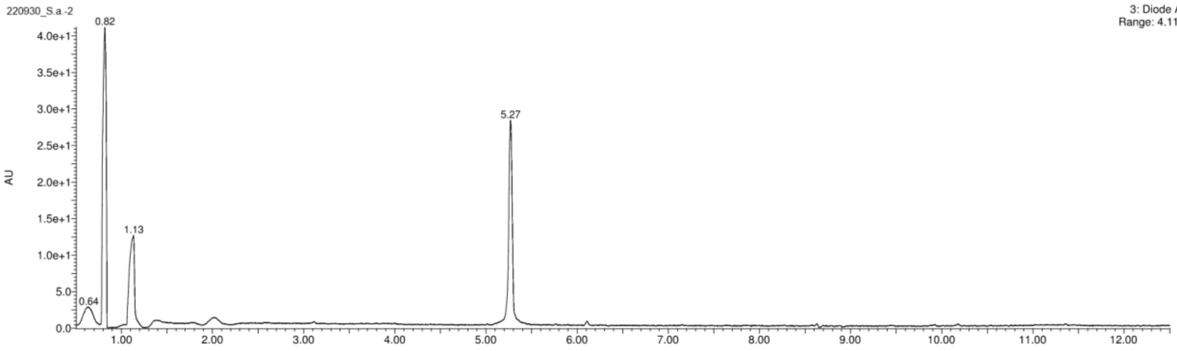

Figure S6. UPLC-PDA chromatogram of compound 2.

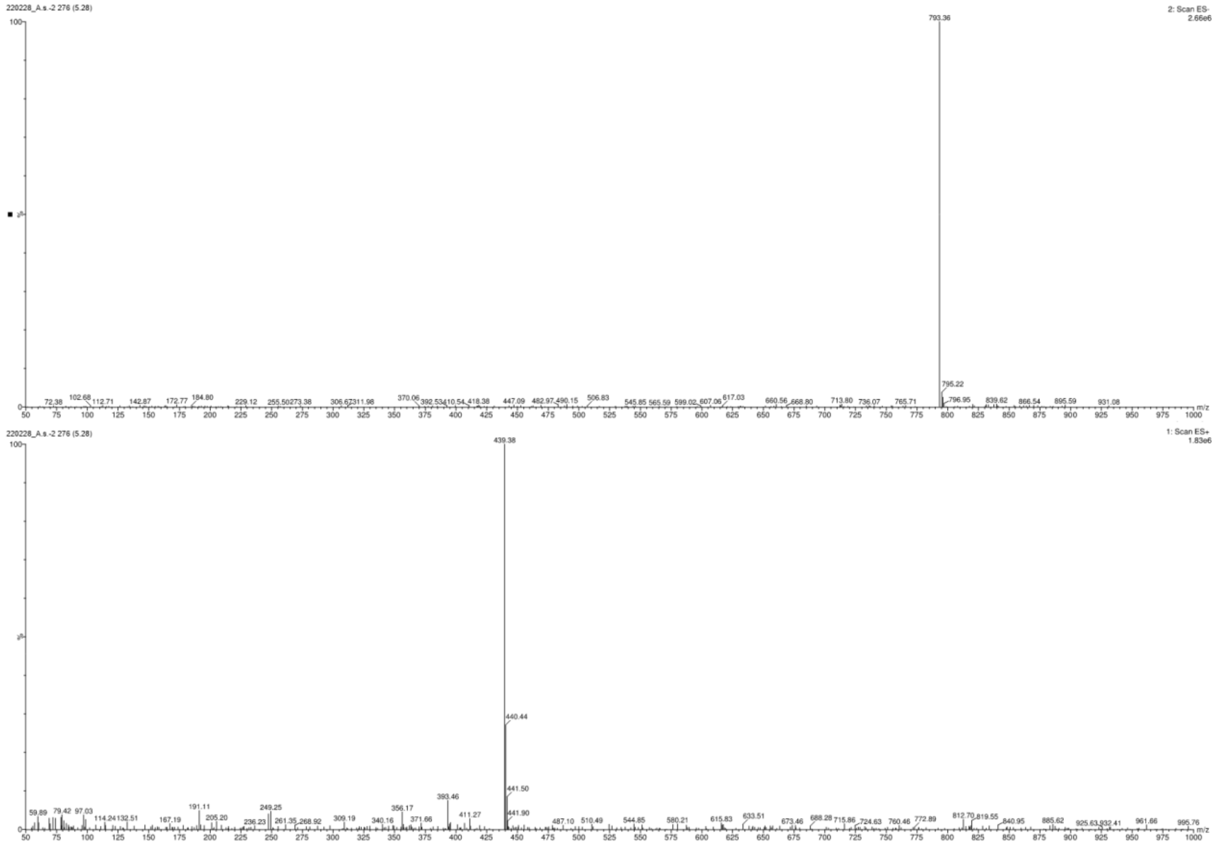

Figure S7. ESI-QTOF-MS (negative and positive ion mode) of compound 2.

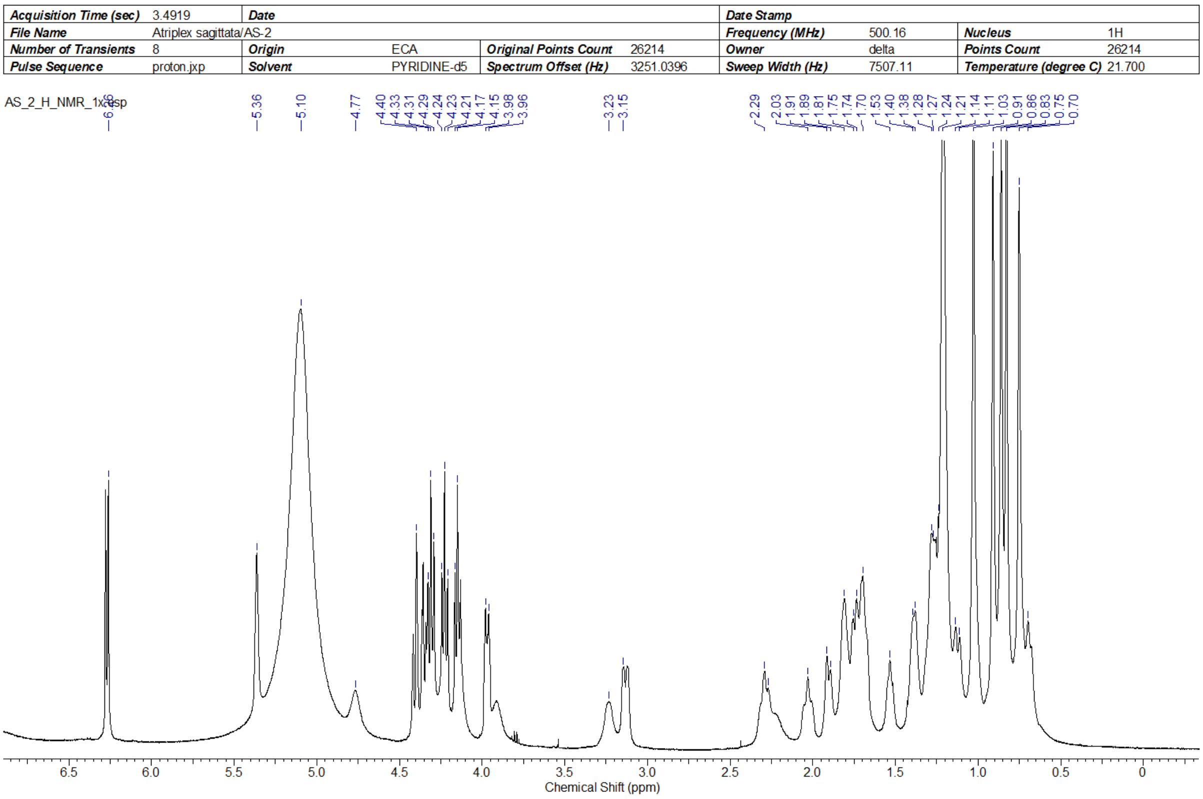

Figure S8. The ¹H NMR (500 MHz, pyridine-d5) spectrum of compound 2.

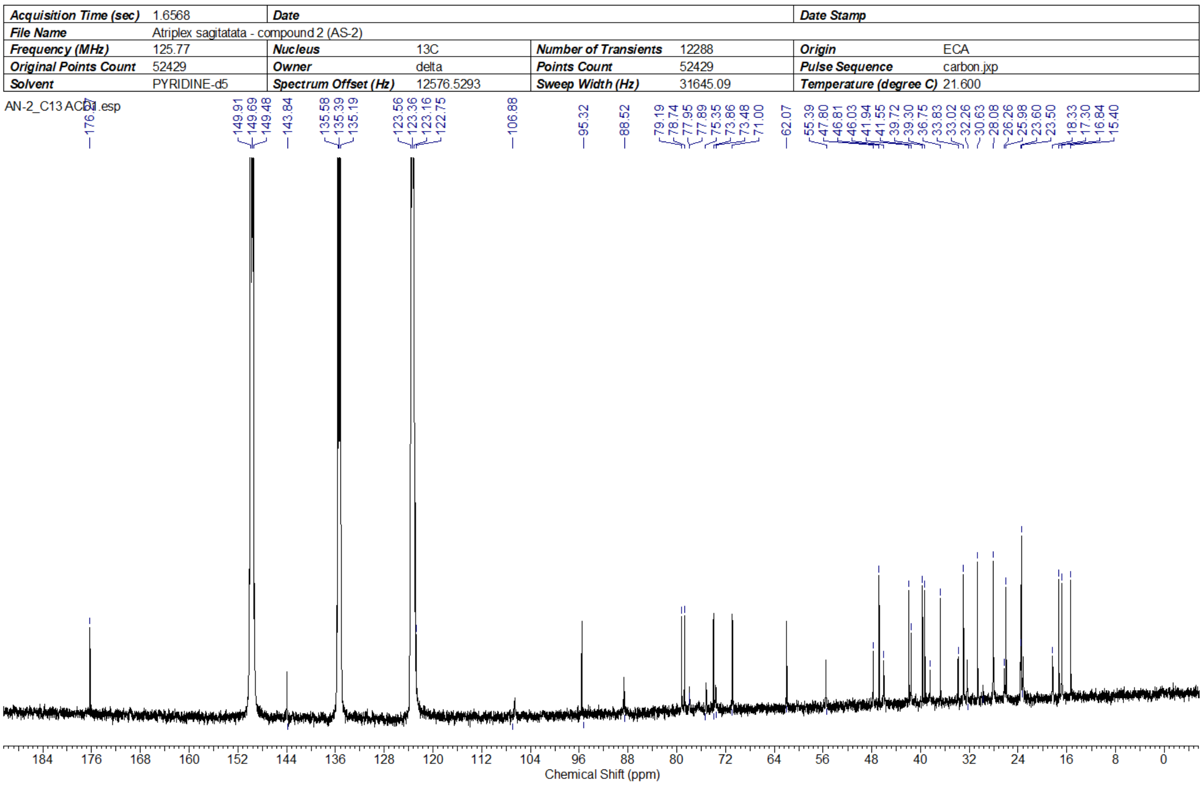

Figure S9. The ¹³C NMR (125 MHz, pyridine-d5) spectrum of compound 2.

Table S5. ^{1}H (500 MHz) and ^{13}C (125 MHz) NMR spectral data (δ ppm) for compound **1** and **2** (pyridine-d5).

	Compound **1**		Compound **2**	
**No.**	**δC**	**δH (*J* in Hz)***	**δC**	**δH (*J* in Hz)***
**1**	38.41	1.37; 0.79	38.48	1.28; 0.75
**2**	26.29	2.22; 1.78	26.26	2.29; 1.81
**3**	88.69	3.26	88.52	3.23
**4**	39.32		39.30	
**5**	55.51	0.73	55.53	0.70
**6**	18.32	1.43; 1.23	18.33	1.38; 1.21
**7**	33.04	1.40; 1.23	32.92	1.40; 1.24
**8**	39.54		39.72	
**9**	47.81	1.56	47.81	1.53
**10**	36.77		36.75	
**11**	23.52	1.83	23.49	1.81
**12**	122.22	5.41	122.75	5.36
**13**	144.58		143.96	
**14**	42.05		41.94	
**15**	29.81	2,09; 1.19	29.81	2.27; 1.14
**16**	22.77	2.07; 1.92	23.23	2.03; 1.91
**17**	46.59		46.81	
**18**	41.72	3.23	41.55	3.15
**19**	46.27	1.76; 1.26	46.03	1.74; 1.21
**20**	30.82		30.62	
**21**	34.07	1.37; 1.15	33.83	1.27
**22**	33.02	1.99; 1.19	32.37	1.80; 1.70
**23**	28.07	1.32	28.07	1.21
**24**	16.83	0.91	16.84	0.91
**25**	15.31	0.78	15.40	0.75
**26**	17.23	0.91	17.30	1.03
**27**	26.05	1.26	25.98	1.21
**28**	180.04		176.27	
**29**	33.16	0.91	33.02	0.86
**30**	23.61	0.95	23.60	0.83
3-*O*-*β*-D-GlcA				
1	106.59	4.83	106.57	4.77
2	75.24	3.96	75.35	3.98
3	78.04	4.19	77.95	4.17
4	73.44	4.32	73.48	4.23
5	77.98	4.35	77.89	4.29
6	nd		nd	
28-*O*-*β*-D-Glc				
1			95.32	6.26 d (8.0)
2			73.86	4.15
3			78.74	4.21
4			71.00	4.24
5			79.19	3.96
6			62.07	4.40; 4.33

* Overlapping signals are reported without designated multiplicity.
